# Communicating artificial neural networks develop efficient color-naming systems

**DOI:** 10.1073/pnas.2016569118

**Published:** 2021-03-15

**Authors:** Rahma Chaabouni, Eugene Kharitonov, Emmanuel Dupoux, Marco Baroni

**Affiliations:** ^a^Facebook AI Research, 75002 Paris, France;; ^b^Cognitive Machine Learning, ENS - EHESS - PSL Research University - CNRS - INRIA, 75012 Paris, France;; ^c^Institució Catalana de Recerca i Estudis Avançats, 08010 Barcelona, Spain

**Keywords:** efficiency of human language, language emergence in artificial neural networks, color-naming systems

## Abstract

Words categorize the semantic fields they refer to in ways that maximize communication accuracy while minimizing complexity. Focusing on the well-studied color domain, we show that artificial neural networks trained with deep-learning techniques to play a discrimination game develop communication systems whose distribution on the accuracy/complexity plane closely matches that of human languages. The observed variation among emergent color-naming systems is explained by different degrees of discriminative need, of the sort that might also characterize different human communities. Like human languages, emergent systems show a preference for relatively low-complexity solutions, even at the cost of imperfect communication. We demonstrate next that the nature of the emergent systems crucially depends on communication being discrete (as is human word usage). When continuous message passing is allowed, emergent systems become more complex and eventually less efficient. Our study suggests that efficient semantic categorization is a general property of discrete communication systems, not limited to human language. It suggests moreover that it is exactly the discrete nature of such systems that, acting as a bottleneck, pushes them toward low complexity and optimal efficiency.

## Significance

Color names in human languages are organized into efficient systems optimizing an accuracy/complexity trade-off. We show that artificial neural networks trained with generic deep-learning methods to play a color-discrimination game develop color-naming systems whose distribution on the accuracy/complexity plane is strikingly similar to that of human languages. We proceed to show that efficiency and narrow complexity crucially depend on the discrete nature of communication, acting as an information bottleneck on the emergent code. This suggests that efficient categorization of colors (and possibly other semantic domains) in natural languages does not depend on specific biological constraints of humans, but it is instead a general property of discrete communication systems.

Words partition our world into semantic categories. Converging evidence indicates that, while these categories differ widely across languages, they are shaped by universal constraints (1–3). In particular, it has been suggested that semantic categorization evolves to support efficient communication (4). Humans develop naming systems to talk about their experience under two competing pressures: “accuracy maximization” (words should encode precise information about their referents) and “complexity avoidance” (preventing unwieldy languages). At an extreme, a maximally accurate system would have a different term for each perceptual or mental experience. At the other, a maximally simple system would use only one term to refer to all experiences, completely hindering communication.

Actual human naming systems are efficient in the sense that they optimize the accuracy/complexity trade-off. More generally, since the foundational work of Zipf (5), a similar trade-off between precision and simplicity has been observed in many areas of language (6).

Zaslavsky et al. (7) formalized the measurement of naming-system efficiency within the general information–theoretic framework of the Information Bottleneck (IB) (8) (see also the closely related rate-distortion theory framework in ref. 9). A system is deemed efficient if it reaches the maximum possible accuracy for a given complexity. In the IB framework, both accuracy and complexity are computed in a communication model where an idealized Speaker aims to communicate a meaning to an idealized Listener. Accuracy is then inversely related to the cost of a misinterpreted meaning, while complexity measures the quantity of information needed to convey the meaning. The IB efficiency of a system is effectively visualized in plots (see Fig. 3). The black curve in Fig. 3 represents the theoretical limit: no system of a certain complexity (horizontal axis) can have accuracy (vertical axis) above the curve. Hence, according to IB, a system is optimal if it lies on the curve. Equipped with this framework, Zaslavsky et al. (7) demonstrated that color-naming systems (4, 10, 11) are notably close to the theoretical limit and hence efficient in a quantifiable way.

IB theory is agnostic about where on the theoretical-limit curve a system should lie. Degenerate systems lying at the extremes of the curve, and expressing each referent with a different term or all referents with a unique term, are also efficient according to this theory. However, such systems are not attested. Instead, real color-naming systems approximate a small range of possible optimal solutions, avoiding the extremes, and in particular high-complexity trade-offs (7). This avoidance of complexity extremes has been observed more broadly in studies of categorization and naming across many semantic domains (4, 12–14).

We study the efficiency of color naming from a different perspective. We compare natural language systems with those emerging from the interaction of modern neural networks (NNs) faced with a color-communication task. Artificial NNs trained with deep-learning methods (15) have recently been used to study human (neuro)cognition in many fields (e.g., refs. 16–19), including color naming (20, 21). Traditional simulations in cognitive science are specifically designed to assess how certain factors of interest affect system behavior by developing ad hoc models, an approach illustrated by Baronchelli et al. (22) and Loreto et al. (23), in the domain of color naming, and applied by Carr et al. (24) to the study of complexity/accuracy trade-offs in semantic categorization. Deep networks, however, are high-performance general-purpose learners, independently developed for engineering purposes, with no claims of cognitive plausibility concerning their architecture or learning process. In this respect, they might be best seen as complex “animal models” (25, 26). The main interest lies in whether the emergent behavior of these powerful mechanisms mirrors nontrivial properties of human behavior (27). If it does, we can entertain the intriguing hypothesis that the specific converging human and deep-network patterns we observe have common roots. We can moreover directly intervene on the artificial organisms (more easily so than we can on humans), in order to causally assess how different components affect their emergent behavior.

Specifically, we show that, when two deep learning-trained NNs play a simple color discrimination game, they develop naming systems that closely match the distribution of human languages on the IB plane, showing both efficiency maximization and complexity control (Fig. 3). The use of human-like artificial systems emerges without imposing ad hoc constraints favoring efficiency or limiting complexity on the training procedure. Having observed the systematic emergence of efficiency and complexity reduction in the NN systems, we proceed to test the hypothesis that these properties crucially depend on the bottleneck imposed by the discrete communication channel. Indeed, as we let NNs exchange messages that are increasingly more continuous, their naming systems become more complex, and, eventually, no longer efficient. Varying the degree of color-discrimination granularity required to play the game affects the complexity of the emergent systems, but not efficiency, and only within the range of attested human variation. NN capacity only affects the complexity of the system in function of discreteness of communication.

The emergence of efficient and reasonably simple semantic categorization is not specific to human language but might generally arise in cognitive devices exchanging discrete messages about their world. Discreteness of communication plays a central role in the emergence of efficient and low-complexity naming systems among our artificial agents, raising intriguing questions about the role of discreteness in human language.

## Color-Naming Task

### Stimuli.

**Fig. 1.**
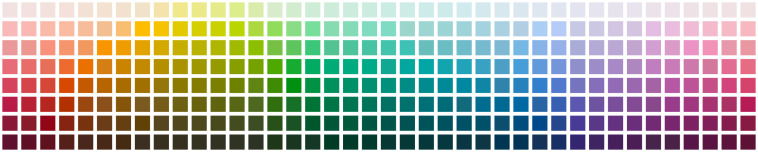
The 330 WCS color chips. Rows correspond to equally spaced lightness values and columns to equally spaced Munsell hues. Each stimulus is at the maximum available saturation for that hue/lightness combination.

Following prior work (4, 7, 28), we use the World Color Survey (WCS). The WCS contains the names of 330 color chips (Fig. 1) in 110 languages of nonindustrialized societies (29). We represent each color stimulus as a three-dimensional vector in CIELAB space (a color space designed to approximate human vision). In particular, we measure color similarity based on Euclidean distance in CIELAB, as it correlates with human perceptual sensitivity (7).

### Discrimination Game.

**Fig. 2.**
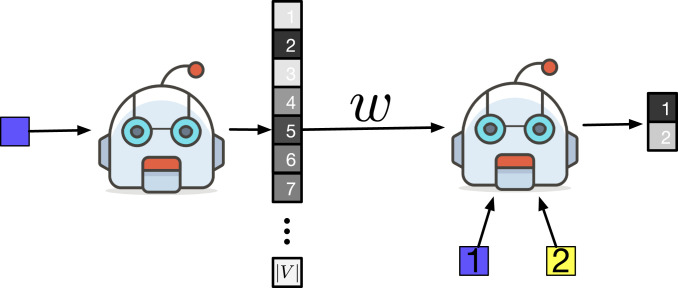
A successful round of the discrimination game. A chip c is drawn from a uniform distribution and fed to Speaker. Speaker outputs a probability distribution p(W|c) over its vocabulary of size |V|. Here, a probability is mapped to a color according to a gray gradient (with darker colors representing higher probabilities). A word w is sampled from p(W|c) and fed to Listener. Finally, Listener—given w, the target chip (in position 1 in this illustration), and a distractor chip (in position 2 in this illustration)—assigns a probability to both positions, representing its guess about the position of the target (in this illustration, Listener correctly assigns a higher probability to the target position).^¶^

We implement a classic discrimination game (30) played by 2 NN agents, Speaker and Listener. Speaker receives a target color $c_{t}$ from the palette and sends one word w from its vocabulary V to Listener. Speaker chooses the word from a fixed vocabulary of size |V| = 1,024. As |V| is larger than the number of colors (330), it is always possible, in principle, for Speaker to use a unique word to denote each distinct color. Given w and two distinct colors, $c_{t}$ and a distractor $c_{d}$, Listener must predict the target. The agents succeed if Listener guesses the correct target (as in Fig. 2).

As in previous work (4, 28), we assume a uniform prior distribution p(c) over target colors. In *SI Appendix*, *Supporting Information Text*, *3. Salience-Weighted Source Distribution*, we test an alternative nonuniform prior (31), and the results still hold.

The game is implemented in EGG (32). Further details are in *Materials and Methods*.

### Discriminative Need.

Despite the presence of universal tendencies (10, 33, 34), color-naming variance is also observed (35, 36). Prior studies hypothesized that such variance depends on distinct frequencies of occurrence of colors across communities (31, 37). In lack of data capturing these differences, we explore a complementary source of variation, that is easier to model computationally. We hypothesize that different cultures have different discriminative needs. Intuitively, highly industrialized societies might need to distinguish between subtly different color shades characterizing different goods, whereas nonindustrialized societies can rely on coarser distinctions. As indirect evidence, Gibson et al. (31) reported that, in the nonindustrialized Tsimané community, color terms are “only used to discriminate between familiar artificial objects.” Since in a nonindustrialized community, there is relatively low variety of artificial objects, discrimination need will be low. In English, instead, speakers systematically use color terms to discriminate between objects of all kinds (31).^†^

Concretely, we define discriminative need as the minimum allowed Euclidean distance between targets and distractors in CIELAB space. Agents trained with small minimum target-distractor distance, $dist_{min}$, simulate communities with high discriminative need; the ones trained with large $dist_{min}$ represent communities with low discriminative need. We quantify $dist_{min}$ in terms of the *n*th percentile in the list of pairwise distances between the 330 distinct color chips. For example, with percentile = 50, for a given target color $c_{i}$, a distractor $c_{j}$ is sampled uniformly among candidate colors such that

$$dist\left(c_{i},c_{j}\right)\geq med\left(\left\{dist\left(c_{k},c_{l}\right);k,l\in \left\{1..330\right\},k>l\right\}\right),$$ [1]

where med is the median function and dist is the Euclidean distance in CIELAB space. Note that larger percentiles correspond to games requiring less granular discrimination. We provide examples in *SI Appendix*, *Supporting Information Text*, *1. Example of Nearest Target-Distractors for Different Percentiles*.^‡^

### Speaker Word Distributions.

Just like in natural language, we allow fuzzy naming: the same color might be, in different occasions, denoted by different words. To estimate the probability distribution P(w|c) associated to a color chip c, we sample 25 words with replacement from Speaker after convergence.^§^ For instance, since Speaker’s outputs form a categorical distribution over V, if this distribution is a Dirac, the resulting set of 25 samples will correspond to a unique word. At the other extreme, if Speaker has no confidence about c’s category, we might get 25 distinct words equiprobably naming c.

### Evaluating the Accuracy/Complexity Trade-Off.

To compareNN and human naming systems, we adopt the communication model of Zaslavsky et al. (7), keeping the same notation. U represents the set of world’s objects, in our case, the set of colors; W represents the set of words; and M represents the set of Speaker’s meanings. We assume that a NN Speaker, similarly to what is conjectured for humans (4, 7), internally represents each target color chip c as a Gaussian distribution m∈M over U centered at c and defined upon CIELAB color similarity. That is, for a given target chip c, Speaker constructs an internal representation m(c) reflecting its belief about the color chip it wishes to communicate to Listener. The Gaussian m(c), of mean c, is then only parameterized by variance $\sigma^{2}$, that informs about the Speaker’s (un)certainty about its belief. Concretely, an m(c) with low variance, reflecting a certain belief, would only cover c and few neighboring chips according to the CIELAB space (e.g., slightly darker and lighter chips). Similarly to Zaslavsky et al. (7), we set $\sigma^{2}=64$ for all target chips. Note that M is only introduced to compute the accuracy and complexity measures below, and it plays no direct role in the discrimination game.

In the framework by Zaslavsky et al. (7), the complexity of a naming system is quantified by the number of bits of information required for expressing the intended meanings. As shown by Zaslavsky et al. (7), this is measured by the mutual information, I(M;W) between M and W.

Also following Zaslavsky et al. (7), we use I(U;W) to measure the accuracy of a naming system. The latter measure is inversely related to the Kullback–Leibler divergence between Speaker and Listener meanings. That is, the better Listener is at reconstructing Speaker’s meaning, the larger I(U;W) is.

The theoretically optimal trade-offs between complexity and accuracies are approximated by minimizing the IB objective function:

I(M;W)−βI(U;W)  s.t. β≥1, [2]

where β is the trade-off parameter determining the relative weight a system will attribute to complexity avoidance vs. accuracy maximization. Both complexity and accuracy are quantified by mutual information terms. However, the IB objective minimizes the first term (lowering complexity) and maximizes the second (increasing accuracy; note the minus sign preceding the second term in Eq. **2**), two constraints that will be in contrast.

**Fig. 3.**
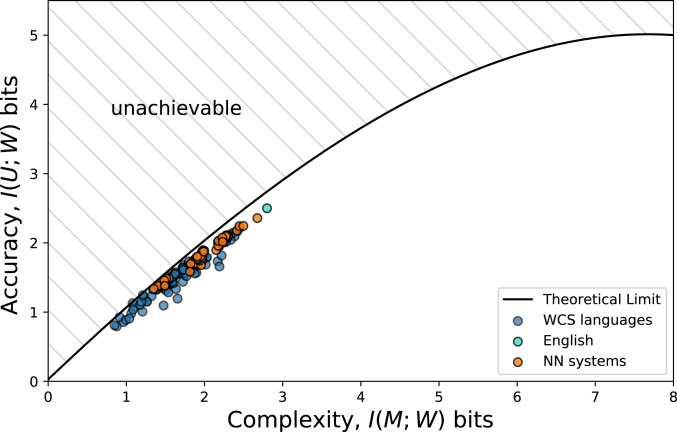
Human (blue circles) and NN (orange circles) color-naming systems on the information plane. English (light blue circle) is not in WCS, but it is approximated relying on Zaslavsky et al. (*SI Appendix*, figure S7 in ref. 7). The IB curve (black line) defines the theoretical limit on accuracy given complexity. All color-naming systems achieve near-optimal efficiency.

To minimize Eq. **2** for a fixed β, we look for the set ${\left\{P\left(w_{i}|c_{j}\right)\right\}}_{i,j}$, where j∈[1,330] and i∈[1,K], with K a variable to optimize. To get the theoretical-limit curve shown in Fig. 3, we repeat this procedure for each β, as described in *Materials and Methods*.^#^ Refer to Zaslavsky et al. (in particular, *Bounds on Semantic Efficiency* in the main text of ref. 7 and *SI Appendix*, section S1.3 in ref. 7) for more details about definitions and derivations.

The farther a system is to the theoretical-limit curve, the less efficient it is. To quantify the inefficiency of a system s, characterized as a point on the accuracy/complexity plane (Fig. 3), we introduce the Inef score:

$$Inef\left(s\right)=\underset{\beta }{\min}\left\{{\left\|s-s_{\beta }^{*}\right\|}^{2}\;s.t.\;\beta \geq 1\right\},$$ [3]

where $s_{\beta }^{*}$ are the coordinates of the optimal naming system (on the theoretical-limit curve) for a fixed β.

## Experiments and Results

### Human vs. NN Naming Systems.

To simulate communities with different needs, we run the discrimination game varying minimum target-distractor distance, defined in terms of percentile of nearest distractor (see *Discriminative Need*). With percentile < 20, target-distractor pairs are too close, and agents fail to converge. Above percentile = 80, there are no distractors sufficiently distant to any given target. We hence played the game with percentile∈{20,30,40,50,60,70,80}, resulting in 60 successful games in total. Control experiments are in *SI Appendix*, *Supplementary Information Text*, *5. Encouraging the Emergence of a Two-Word System during Training*.

Looking at how human and NN naming systems spread along the IB line in Fig. 3, we can make two striking observations. First, NN systems lie near the theoretical IB limit just like human languages do. *SI Appendix*, *Supplementary Information Text*, *4. Efficiency: Comparing Human vs. NN Systems, and Actual vs. Rotated Systems* shows that NN system inefficiency (Eq. **3**) falls within the human range. Second, both human and NN systems lie on a narrow segment of the curve. While this segment does not include the minimal values, it is still clearly tilted toward the low-complexity end of the curve. Note that minimum complexity would be achieved by a system with a single color term. As this makes no sense, we do not expect minimum-complexity systems to emerge. Intriguingly, neither WCS nor NN systems include two-word codes (which are exceedingly rare in natural languages in general) (39). *SI Appendix*, *Supplementary Information Text*, *5. Encouraging the Emergence of a Two-Word System during Training* shows that, even when we manipulate the game so that agents could achieve perfect discrimination with two words only, they minimally converge to a three-word system. We have no explanation for why two-word systems are avoided. Still, both WCS and NN systems are clearly coming much closer to the lower end of the complexity scale than to the upper bound.^∥^

In sum, standard NNs trained on the discrimination game develop systems that support efficient communication (i.e., are close to the IB curve) while preferring low complexity, similarly to human color-naming systems. Our focus here is on the IB trade-off. However, the way in which NN systems accomplish this trade-off is not radically different from that of human languages. *SI Appendix*, *Supplementary Information Text*, *10. Direct Comparison of Color Space Partitions* presents a detailed comparison of color partitioning in human and NN naming systems, highlighting partial differences but also important commonalities, in particular, in terms of the convexity of regions corresponding to distinct color names (see also *SI Appendix*, Fig. S12 for qualitative comparison between both systems).

### Effect of discriminative need.

**Fig. 4.**
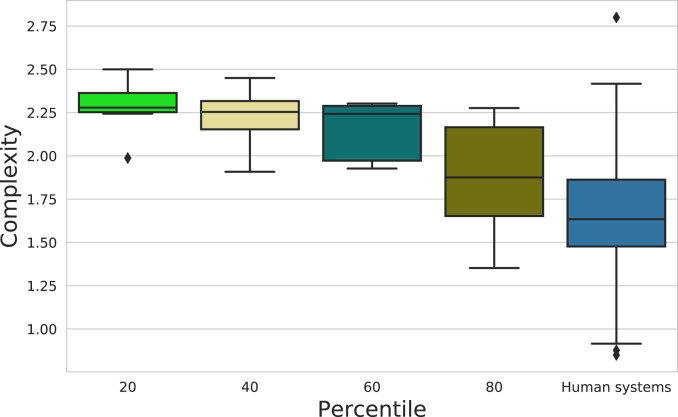
Complexity distributions of NN systems across different discriminative needs (human distribution included for comparison). There is a decreasing trend in complexity when increasing percentile (*P* = 0.004; Kruskal–Wallis). Pairwise differences are not significant when evaluated with Bonferroni-corrected Mann–Whitney–Wilcoxon.

Fig. 3 shows the NN systems resulting from exploring the full range of possible percentile values (the parameter controlling discriminative need). While all systems are efficient, we observe some variability in complexity (within the [0.84,2.8] range), that might be due to different discriminative needs. This is confirmed by Fig. 4, which shows NN naming system complexity in function of percentile. Smaller percentile values (requiring more granular discrimination) make systems more complex. Still, this trend is gradual with no significant pairwise differences, suggesting the need for distant discriminative needs to observe a significant difference in systems’ complexity. Furthermore, NN systems’ complexity remains within human-range complexity when exploring the full range of percentile values. Interestingly, Fan et al. (14) showed, in the context of visual communication, that humans are also sensitive to discriminative need and adapt the complexity of their communicative system accordingly.

Thus, discriminative need (or related environmental/societal pressures to make more/less granular distinctions) could account for the range of complexity variation we observe in NN and human naming systems (and that might be somewhat underestimated by the WCS sample). However, alone, it does not explain why the range of observed systems is so narrow.

### Preference for low complexity.

Both human and NN systems show much lower complexity than what could be found in an optimal naming system by systematically varying the trade-off parameter β∈ [1,+∞[.** The attested systems all occur within a small segment corresponding to β∈ ]1, 1.14].

One might conjecture that more complex codes do not evolve simply because the attested ones are sufficiently granular to support all required discriminations. For our NN agents at least, this is not the case, as they systematically fail to achieve 100% success in the discrimination game, which would instead be possible with more complex systems. To illustrate the latter, we generate additional naming systems by partitioning the color space using the “fuzzy c-means” (FCM) soft clustering algorithm (40), treating cluster labels as color names. We obtain different systems by varying the number-of-clusters hyperparameter. We then play the discrimination game with Speakers and Bayesian Listeners that use p(w|c) distributions derived from the soft clustering solutions.

The FCM-based agents can reach 100% communication success at all percentiles. However, this comes at the cost of higher complexity. Table 1 compares, for each percentile, the 100% successful FCM system with lowest complexity to the NN system with highest success rate. In all cases, NNs came up with systems that are considerably less complex but that also fail to reach perfect discrimination success.^††^ We conclude that the low complexity of NN systems cannot be explained by lack of sufficient communicative pressure toward more complex solutions. We explore next other possible sources of low-complexity-preference.

**Table 1.** Complexity and success rate (game accuracy after training) of FCM-based and NN systems in function of the game percentile parameter

	min complexity	Complexity	Success rate
Percentile	FCM	Best NN	Best NN
20	5.39	2.50	95.45%
30	4.34	2.28	96.97%
40	4.01	2.23	95.76%
50	3.75	2.68	98.79%
60	3.44	2.17	96.97%
70	3.39	2.30	97.56%
80	3.12	2.24	98.78%


FCM success rate is always 100%. For FCM, we report minimal complexity among fully successful solutions. For NN, we report complexity and success rate of the system achieving highest success rate.

### Roots of Efficiency and Complexity Avoidance.

Building on recent work (41), we explore the idea that the discrete nature of the communication channel acts as bottleneck on the amount of information that the agents are able to transmit, leading them to establish efficient and low-complexity naming systems. Another natural bottleneck could be agents’ capacity. Perhaps, the “neural power” of our NNs does not suffice to develop more complex languages. We show next that channel discreteness plays a fundamental role in complexity reduction, whereas NN capacity only matters insofar as it allows the agents to further simplify the code in presence of a discrete channel.

### Effect of channel discreteness.

We fix percentile = 50 and explore different training regimes ranging from a fully discrete setup to a virtually continuous one, relying on two commonly used methods to train deep networks in language emergence scenarios (e.g., refs. 42 and 43; also see *Materials and Methods*). The REINFORCE (RF) algorithm uses fully discrete symbol transmission during both training and evaluation. The Gumbel-Softmax (GS) method is fully discrete at evaluation time, but it estimates symbol probabilities through a smooth approximation during training. At training time, discrete symbols are approximated by continuous vectors with most of the mass concentrated around a single value. The “peakiness” (and thus discreteness) of this approximation is controlled by the temperature parameter τ. The lower the τ, the peakier the vector (practically converging to a discrete “1-hot” encoding for low τs). We explore τ∈{1,5,10}, corresponding to increasingly smoother communication channels.

Settings with less smooth channels, and in particular fully discrete RF, are harder to train. Hence, we launch 60 runs for each GS setting and 180 for RF. In *SI Appendix*, *Supplementary Information Text*, *6. Discreteness and Success Rate*, we discuss the relation between channel smoothness and successful convergence, arguing that the high failure rate of more discrete settings is due to a higher complexity-reduction pressure.

**Fig. 5.**
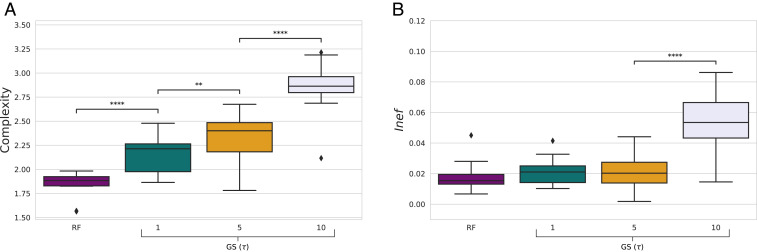
Complexity and inefficiency of NN color-naming systems trained with REINFORCE or GS with different τs. Pairwise differences evaluated with Bonferroni-corrected Mann–Whitney–Wilcoxon. *P < 0.05; **P < 0.01; ***P < 0.001; ****P < 0.0001. Differences that are not significant are not marked.

Fig. 5*A* shows that agents trained with RF (thus, in the completely discrete setting) develop significantly less complex systems compared to the ones trained with GS. Within GS, lower τ (more discreteness) leads to simpler codes. With more complexity, smoother systems also become less efficient, an effect that is clear with the highest τ=10 (Fig. 5*B*).^‡‡^ In *SI Appendix*, *Supplementary Information Text*, *9. How Are Color-Naming Systems (In)efficient?*, we study one concrete way in which these systems are inefficient, comparing them with complex but still efficient NN systems resulting from high discriminative need.

### Effect of agent capacity.

Only Speaker capacity has a significant impact on complexity and only with a discrete communication channel. Interestingly, larger Speakers further reduce the complexity of the emerging naming system (*SI Appendix*, Fig. S9). As further discussed in *SI Appendix*, *Supplementary Information Text*, *8*. *Impact of Agent Capacity*, a reasonable interpretation for this pattern is that, when the channel is discrete, transmitting information is difficult. Consequently, a “smarter” Speaker will use its extra computational power to come up with an encoding that allows it to transmit even less bits through the channel while maintaining reasonable accuracy. Thus, the agents’ capacity experiments further confirm the importance of the discrete-channel bottleneck for complexity minimization.

## Discussion

We have shown that NNs trained to play a color discrimination game develop naming systems whose distribution on the accuracy/complexity trade-off plane strikingly resembles that of human languages. We obtained this result using game success as the sole training signal, without imposing any constraint on the emergent code, except that it had to consist of single discrete symbols. A very recent study by Kågebäck et al. (21) reports that deep NN agents trained with generic techniques to play a color-naming game strike a similarly human-like complexity/accuracy trade-off, despite important differences between their game and ours (in their setup, the Listener receives only the message as input, and it has to reconstruct the color chip seen by the Speaker), different methods to derive a discrete protocol, and different factors modulating the trade-off (the complexity cline, in their experiments, depends on different amounts of noise added to the communication channel). This constitutes important converging evidence that deep-network communication tends to naturally optimize the accuracy/complexity trade-off, independently of the specifics of the simulations.

We observed, in particular, that the networks developed “low-complexity” systems, again in accordance with natural language data. We then looked for the source of this low-complexity pressure in NN systems. Building up on a recent study reporting similar results in artificial tasks (41), we showed that the presence of a discrete communication bottleneck plays a crucial role. As we relax discreteness, the emergent naming systems become complex beyond what is attested in human language, and, eventually, significantly inefficient.^§§^

In the last few years, much evidence for the efficiency of human languages in general (6) and semantic categorization in particular (4) has been accumulated. Yet, we still lack a full scientific understanding of “why” language is efficient. Our results provide two contributions relevant to this question. First, since efficiency and complexity avoidance also characterize the code evolved by communicating NNs, these factors cannot be explained away by least-effort factors specific to biological agents. Second, the fact that NNs exchange a discrete signal is crucial. Discreteness is a striking, possibly unique characteristic of human language (44, 45), often adduced as a precondition for the combinatorial infinity of expression that characterizes it (46). Our finding suggests that it might also be responsible for the efficient nature of semantic categorization (and possibly language at large). We do not have direct evidence on how the language of our ancestors became discrete and on how this affected the structure of semantic categorization. However, our computational results pave the way for experiments with contemporary humans, exploring how a continuous/discrete transition in communication systems affects the nature of information exchange. With human subjects, we might not have a direct equivalent of the GS temperature parameter. We can however build on a strong tradition of experimental semiotics studies using continuous signals, such as drawings, whistles, and nonconventionalized gestures, and sometimes reporting a tendency to discretize the signals as systematic communication strategies emerge (14, 47, 48). By using this framework, we should be able to design experiments that probe a causal relation between discreteness and communicative efficiency, ultimately strengthening our understanding of the roots of efficiency in language.

## Materials and Methods

### Human Languages.

We used the WCS database (www1.icsi.berkeley.edu/wcs/). Two languages with extremely sparse information (judgments from 1 speaker only for at least some chips) were removed, resulting in 108 analyzed languages. English, which is not in WCS, was approximated based on the relevant figures from the study by Zaslavsky et al. (7).

### Agent Architecture and Training.

Both agents are feed-forward NNs. Speaker contains 3 hidden layers, each of size 1,000 and with leaky-ReLU (rectified linear unit) activations. For each color, the Speaker’s output layer defines a Categorical distribution over its vocabulary V. Listener is modeled as a linear layer of hidden size 5. The impact of agents’ capacity on results is discussed in *SI Appendix*, *Supplementary Information Text*, *8. Impact of Agent Capacity*.

Training NNs to communicate through a discrete channel is nontrivial, as we cannot backpropagate into the Speaker through this bottleneck. We use two methods commonly employed in the deep agent language emergence literature: 1) GS relaxation (e.g., ref. 43) and 2) REINFORCE (e.g., ref. 42)) (in both cases, Listeners’ gradients are obtained with standard backpropagation). We plug the obtained gradient estimates into Adam (49).

### GS.

Samples from the GS distribution (50, 51) approximate those from a Categorical distribution through a reparameterization trick, thus enabling gradient-based training. Let us denote $\sigma :R^{n}\to R^{n}$ the standard softmax function. To get a sample that approximates an n-dimensional categorical distribution with probability p, we draw $g=\left[g_{1},\ldots ,g_{n}\right]$, where for each i, $g_{i}∼$ Gumbel(0,1) and use it to calculate y such that:

$$y=\sigma \left(\frac{g+log\;p}{\tau }\right),$$ [4]

where τ is the temperature hyperparameter. As τ tends to 0, the samples get closer to one-hot, making communication more discrete; as τ→+∞, the samples tend to uniform, resulting in smooth communication. At training time only, we use the relaxed samples as messages from Speaker, making the entire Speaker/Listener setup differentiable. We look at the impact of τ on Speakers’ output distribution in *SI Appendix*, *Supplementary Information Text*, *7. Effect of More/Less Discrete Training on Speakers’ Output Distribution*.

## Reinforce

Following Schulman et al. (52), we sample Speaker’s words and estimate its gradients as follows:

$$E_{i_{s},i_{l}}E_{w∼S\left(i_{s}\right)}\left[L\left(o;t\right)+sg\left(L\left(o;t\right)-b\right)\log P_{\theta }\left(w\right)\right],$$ [5]

where $i_{a}$ are agent’s inputs with a=s if agent is Speaker and a=l if it is Listener. o denotes Listener’s prediction, t denotes the ground-truth, and L denotes the cross-entropy loss function; sg refers to the “stop-gradient” operation. We use the standard running mean baseline b (53, 54) to reduce estimate variance. To achieve more robust convergence, we also adopt the common trick to add an entropy maximization term (55, 56) on Speaker’s words. This could favor higher code complexity, which makes our low-complexity result even more striking.

When not stated otherwise, results are based on GS training with temperature τ = 1. Training consists in letting the agents play the game until their performance converge (this happens, on average, after about 6 million interactions). For each considered setting, we repeat experiments with 20 different random initializations and only focus the analysis on the successful runs. We consider a run successful if, after convergence, the agents have at least a 95% success rate. Following standard practice, success rates are computed in games in which the most likely word is deterministically sampled from the Speaker distribution.

### IB Curve.

We use the Agglomerative IB method (57) with $\beta_{init}=2^{13}$. At each step of the annealing process, we evaluate the IB solution, i.e., P(w|c) for each (w,c) using Iterative IB (57). The latter is an iterative method that alternates between evaluating P(w|c) and m(c) (Speaker’s meaning for each c) until convergence. We refer readers to Zaslavsky et al. (*SI Appendix*, section 1.4 in ref. 7) for more details about this two-step process. When annealing β according to Agglomerative IB, the IB solution is initialized with the one found with the previous value of β. Optimization ends when β=1.

## Supplementary Material

Supplementary File

## Data Availability

The models reported in this paper have been deposited in GitHub (https://github.com/rahmacha/EGG).

## Footnotes

^†^Humans can also handle varying contextual discrimination needs by producing longer or shorter phrasal descriptions (38), a strategy we are not modeling here.

^‡^*SI Appendix*, *Supplementary Information Text*, *2. Random Sampling of Distractors* demonstrates that highly efficient systems also emerge when no percentile is imposed, although the latter never reach our threshold for minimum game accuracy (95%).

^§^A majority of WCS languages contains names elicited from 25 speakers, leading to comparable a sample size for P(w|c) estimation.

^¶^Target and distractor positions are randomly shuffled at each round to prevent Listener from relying on position to succeed at the game.

^#^We only optimize Eq. **2** to calculate the IB bound. NN training is completely distinct and independent from this calculation.

^∥^NN systems are tilted toward the top of the human complexity range. This is probably an artifact of WCS’ focus on nonindustrialized societies. English, the only industrialized-society language in Fig. 3, is more complex than any NN language.

**In practice, distinct optimal systems are only obtained for $\beta \in \left[1,2^{13}\right]$, as all optimal systems with $\beta >2^{13}$ are identical and assign a unique word to each color.

^††^In the few cases in which FCM systems converged to success rates comparable to those of NN systems, FCM systems were on average less complex, suggesting that the relatively high FCM complexity we observe in Table 1 is not due to an inherent tendency of the latter to converge to high-complexity solutions.

^‡‡^The trend toward higher complexity and lower efficiency continues with larger τ. However, above τ = 10, agents rarely succeed at the game, making the interpretation of results difficult.

^§§^Intriguingly, while Kågebäck et al. (21) do not explicitly discuss it, their results also point to a correlation between complexity reduction and discreteness, despite the fact that they control discreteness in a different way, that is, by injecting noise into a continuous channel.

## Change History

March 16, 2021: The license for this article has been updated.
