# Supplementary material for: Communicating artificial neural networks develop efficient color-naming systems
Source: Proc Natl Acad Sci U S A. 2021 Mar 15;118(12):e2016569118. doi: 10.1073/pnas.2016569118 (PMC8000426; doi:10.1073/pnas.2016569118)
Supplement: Supplementary File [file pnas.2016569118.sapp.pdf]

Supplementary Information for

Communicating artificial neural networks develop efficient color-naming systems

Rahma Chaabouni, Eugene Kharitonov, Emmanuel Dupoux and Marco Baroni

Corresponding Author name: Rahma Chaabouni
E-mail: rchaabouni@fb.com

This PDF file includes:

Supplementary text
Figs. S1 to S12
Tables S1 to S3
SI References

Supporting Information Text

1. Example of nearest target-distractors for different percentiles

Figure S1 shows examples of nearest target-distractors in games with different percentile values (recall that percentile is the parameter controlling how close target and distractor can be in CIELAB space). It clearly shows how at lower percentiles agents must discriminate between pairs that are visually close. Percentile 5 is below the level at which NNs successfully converge on a naming system. Indeed, Figure S1a shows that playing the game at this level requires distinguishing between color shades for which even a color-name-rich language such as English would have to resort to phrases. Figure S1b illustrates the percentile 20 game, which is the hardest one at which NNs succeed. We observe that some of the distinctions that need to be made (such as those between the first 3 pairs in the figure) are still quite subtle, for a single-word system at least. Figures S1c and S1d illustrate games requiring mid and low discrimination granularity, respectively.

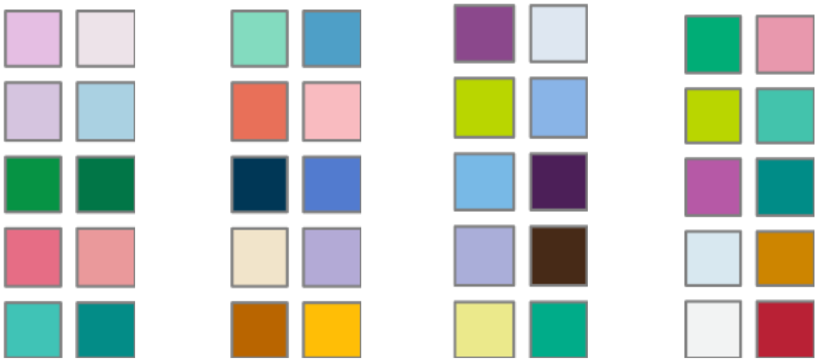

(a) 5 **(b)** 20 **(c)** 50 **(d)** 80

Fig. S1. 5 closest target-distractor pairs for representative percentile values.

2. Random sampling of distractors

As we control discriminative need, we might worry that the efficiency of emergent systems is due to the constraints we impose, favoring solutions where color chips that are close in CIELAB space are named in the same way, rather than being a general property of discrimination-trained NNs. To address this concern, we considered an extreme version of the game, where there is *no* restriction on discriminative need, as both targets and distractors are sampled uniformly and *independently*. This is equivalent to setting discrimination need to the strongest possible pressure ($percentile = 0$).

As it requires discriminating extremely similar colors, for which no language could possibly have distinct words or even phrases, the game is very difficult, and indeed none of 20 runs met our criteria for success ($> 95\%$ discrimination accuracy after convergence). However, 6 runs did reach discrimination success above 90%. Figure S2 compares the corresponding systems to the ones emerging in the standard percentile-constrained games we study in the main text. It is clear that, in terms of efficiency/complexity, the systems emerging from the unconstrained games are comparable to those emerging when controlling discriminative need.

We conclude that our results do not depend on controlling discrimination need through the percentile parameter.

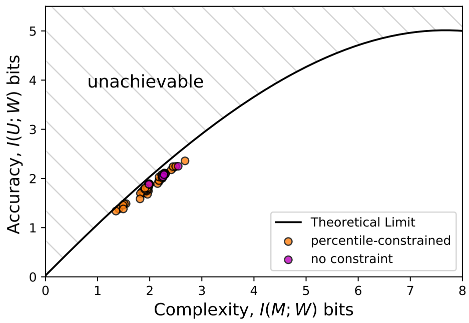

Fig. S2. NN systems emerging from *percentile-constrained* and *no constraint* games plotted on the information plane. The former are those further studied in the main text. The IB curve (black line) defines the theoretical limit on accuracy given complexity.

3. Salience-weighted source distribution

In the main article we assumed a uniform distribution $p(c)$ for chips sampling. To assess the impact of this assumption on the results, we replicate here our main experiment using the salience-weighted (SW) distribution introduced by Gibson et al. (1). This distribution, based on color frequencies in natural images, estimates the probability of a given color c considering the ratio between the frequency with which c appears in objects and its overall frequency (the sum of times c appears both in objects and as background). Gibson et al. (1) originally computed this probability for 80 colors only. To construct a SW prior for the whole WCS palette, we follow ZKRT (2), and use an RBF interpolation with the same parameters. The estimation of this prior is shown in Figure S3.

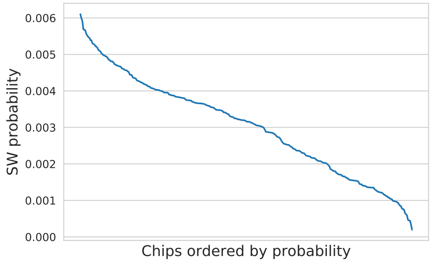

Fig. S3. The estimated salience-weighted (SW) prior over the 330 chips. Chips are ordered by decreasing probability.

We re-run the analysis described in the main paper, but now sampling both targets and distractors according to the SW distribution. Figure S4 confirms that our results do not depend on the uniform assumption made in the main paper. With this alternative skewed input distribution as well (see Figure S3), NN systems are as efficient as the humans ones, and lying just below the same segment of the IB curve.

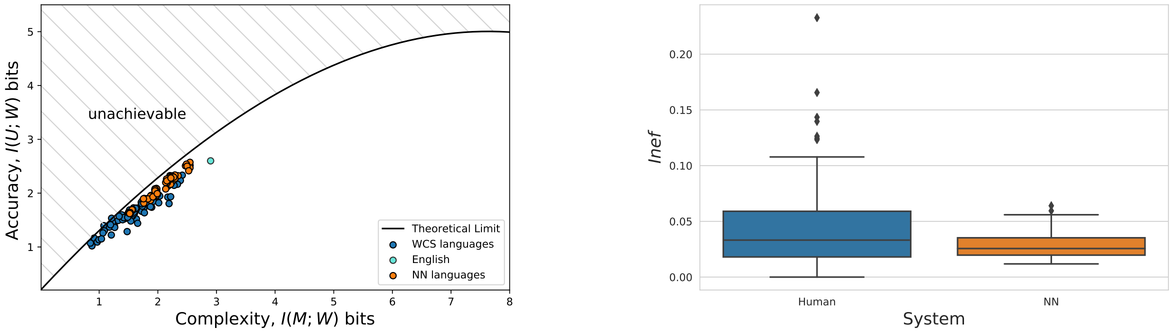

(a) NN and human naming systems in the information plane. The theoretical limit is defined by the IB curve (black line).

(b) Comparing $Inef$ of emergent and human systems (WCS data and English). A t-test fails to detect a significant difference between the two types.

Fig. S4. IB efficiency of human and NN color-naming systems when considering SW input distribution.

4. Efficiency: comparing human vs. NN systems, and actual vs. rotated systems

Both human and NN systems are efficient. Figure S5 shows that the whole distribution of $Inef$ values of NN systems is well-contained within the range of variation attested in human languages.

ZKRT (2) presented a control study in which they compared each human naming system with a set of 39 hypothetical variants obtained by rotating the system along the hue dimension. They showed that the real systems are more efficient than the control set. In this section, we replicate the analysis with human systems and extend it to NN ones.

First, as shown in Figures S6a and S6b, and similar to ZKRT (2), we find that actual human naming systems are on average closer to the theoretical optimal limit: 98% of human languages attain a better trade-off than their rotated counterparts. Interestingly, this pattern is even stronger in NN naming systems, as they *all* achieve a better trade-off compared to any of their hypothetical variants (cf. Figures S6c and S6d).

5. Encouraging the emergence of a two-word system during training

We observed in the main text that the simplest NN systems include 3 terms. To test whether NNs could in principle develop a simpler system, we designed two variants of the discrimination game where we are positive that 100% performance could be

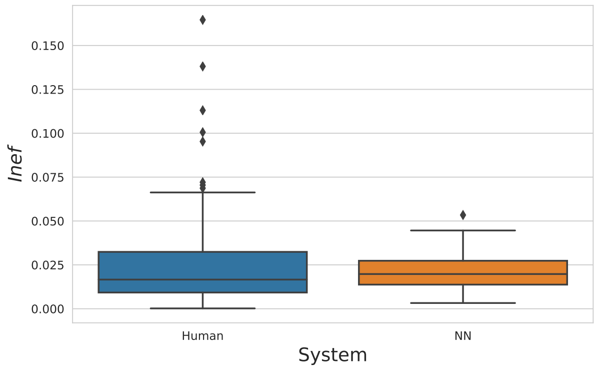

Fig. S5. Comparing $Inef$ of NN and human systems (WCS data and English). A t-test fails to detect a significant difference between the two types.

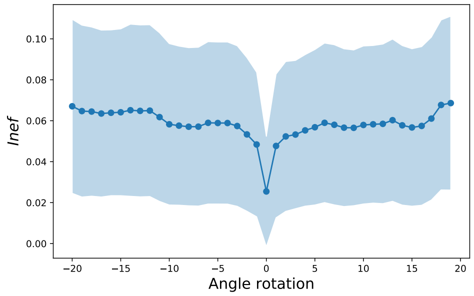

(a) Human systems: $Inef$ of actual WCS naming systems (at rotation$=0$) vs. their rotated hypothetical variants. The continuous curve represents averages across systems for each rotation degree, and the colored region marks standard deviation across systems.

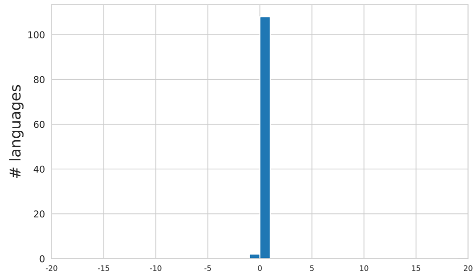

(b) Human systems: Histogram of distribution of most efficient rotation across WCS data and their hypothetical variants. As can be seen, in nearly all cases the highest-efficiency rotation is the 0-rotation, that is, the actual language

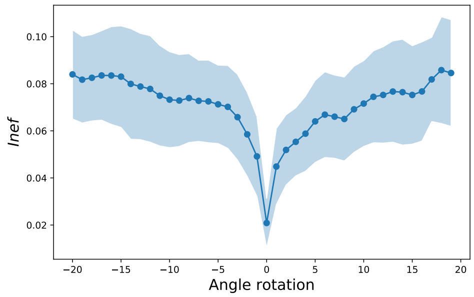

(c) NN systems: $Inef$ of actual NN naming systems (at rotation$=0$) vs. their rotated hypothetical variants. The continuous curve represents averages across systems for each rotation degree, and the colored region marks standard deviation across systems.

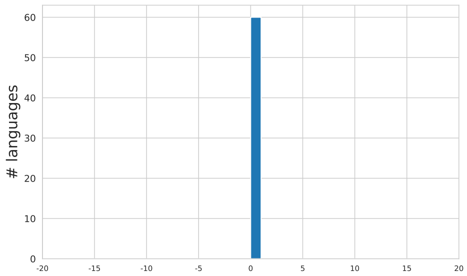

(d) NN systems: Histogram of distribution of most efficient rotation across NN systems and their hypothetical variants. The 0-rotation system (that is, the actual NN system) is the most efficent one in all cases.

Fig. S6. Comparing WCS and NN naming systems with their rotated variants. Rotation 0 corresponds to actual systems.

attained using two color words only.

To construct the first game, we use the FCM clustering algorithm (see main text) to partition the color space into two clusters optimized for minimal intra-cluster distance (FCM is a fuzzy clustering algorithm, but we discretize its outcome to obtain a hard partition). As shown in Figure S7a (left), this leads roughly to a yellow/other distinction. For the second game, we partition the color space into dark and light regions (see Figure S7b (left)). This partition is in line with the basic distinction found in human languages with two color-terms (such as Dani) (3).

In both settings, we ensure that target and distractor always come from the two distinct clusters. To do so, we sample a target color uniformly from the 330 candidate colors. Then, knowing the cluster of the target color, we sample a distractor, also uniformly, from the other cluster. Thus, a system could reach 100% performance by relying on two names denoting the two clusters.

We find that this setup is relatively hard for NNs: only 3/20 runs succeed in each game. Average success rate across the latter runs is at 97%. The corresponding NN systems minimally feature 3 terms, and are more complex than necessary. Concretely, a 2-term system could have complexity 0.87 and 0.99 in the FCM-based and dark/light games, respectively. NNs develop systems with an average (std) complexity of 1.32 (0.22) and 2.13 (0.15), for the respective games. Though NNs use more words than needed, we observe in Figure S7 that their systems stay close to the ground-truth partitions (yellow/other with a supplementary reddish term in Figure S7a, and dark/light, but referring to dark with multiple terms in Figure S7b).

6. Discreteness and success rate

Coherently with the fundamental observation that communicating across a discrete channel is more challenging, which leads to the complexity avoidance effect we discuss in the main text, we also observe that fewer simulation runs are successful when the channel is more discrete.*

Recall that REINFORCE (RF) messages are purely discrete both at training and at testing time, and that, for Gumbel-Softmax (GS), the lower the τ, the closer the approximation to discreteness at training time is. Table S1 shows that success rate is clearly correlated with channel smoothness, with the exception of GS with $\tau = 10$, which is the "smoothest" setting, but also one of the most difficult ones.

*As usual, we consider a run successful if, after convergence, the NNs can correctly communicate about at least 95% of the possible distinct targets.

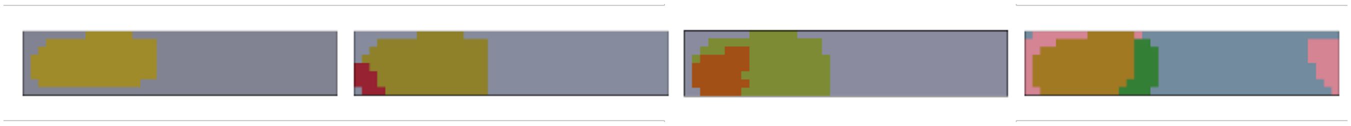

(a) Color space partition obtained with FCM clustering with 2 clusters (left panels), and the 3 successful NN systems trained on a game where targets and distractors are always sampled from the two distinct clusters (next 3 panels).

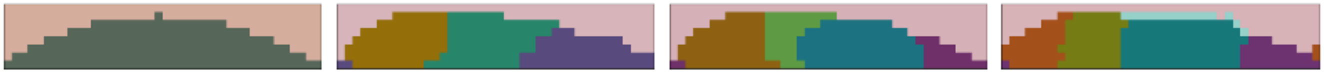

(b) Dark/light partition of the color space (left), and the 3 successful NN systems trained on a game where targets and distractors are always sampled from the two distinct regions (next 3 panels).

Fig. S7. Ground-truth partitions of the space used to design the discrimination games (left) and the 3 corresponding successful NN systems in each game. Cluster colors are obtained by averaging the RGB values of all chips in the cluster.

We conjecture that the low success rate of GS with $\tau = 10$ stems from different reasons than failures in the more discrete settings. In particular, we expect that, in the more discrete settings, complexity of the emergent system after training is systematically *lower* in failed runs, because failures stem from the difficulty of establishing a sufficiently complex protocol through the discrete channel. However, this should not be the case for $\tau = 10$, where complexity should be comparable in failed and successful runs. We verify this hypothesis quantitatively in Table S1 by comparing, for each setting, the complexity of failed and successful naming-systems. We observe that, if successful systems have systematically larger complexity, this difference is only significant when communicating with a discrete(-like) channel (RF and GS with $\tau = 1$). For the remaining settings, there is no significant difference in complexity between successful and failed systems. This supports the claim that failure correlates with lower complexity only when communicating through a more discrete channel. The low success rate of GS with $\tau = 10$ may be explained by the noise introduced in that setting when approximating Categorical samples. Indeed, as mentioned in the main paper, larger τ leads to more continuous, thus noisier, estimation of messages.

Setting	τ	success rate	avg. complexity	
			successful systems	failed systems
RF	-	7.8%	1.60	0.75*
GS	1	46.7%	2.13	1.42*
	5	46.7%	3.06	2.90
	10	20%	3.00	2.81

Table S1. Relation between channel smoothness, success rate and complexity for successful and failed systems. * marks significant differences (t-test, $p < 0.001$).

7. Effect of more/less discrete training on Speakers' output distribution

Recall that, following the human data modeling of ZKRT (2), we let Speaker define an output word probability distribution given each input color, $P(w|c)$. Here, we ask whether this distribution becomes flatter as we train it in an increasingly smoother (less dicrete) setup.

Figure S8 shows the average $P(w|c)$ entropy after training in settings ranging from purely discrete REINFORCE (RF) to Gumbel Softmax (GS) with increasing τ (corresponding to more smoothness during training). Here, entropy measures Speaker uncertainty about which term to use for a certain color input. In the more discrete settings, entropy is approximating 0, which would correspond to a categorical distribution (only one word is produced for each input). As smoothness increases, entropy also increases. However, Speaker is still, on average, quite confident about which term to pick, as entropy is still far from its uniform-probability level.

8. Impact of agent capacity

Informal experimentation showed that, provided that Speaker is powerful enough, the color discrimination task is easily solved by simple Listener NNs. As larger Listeners are harder to train, the main experiments use the simplest possible Listener succeeding at the game, that is, a 1-layer NN with 5 hidden units. In contrast, large Speakers were necessary for game success. We experiment with 3-layer NN Speakers of hidden size 1000. Although simpler Speakers are occasionally able to learn the game, our preliminary experiments showed that the chosen combination led to significantly more successful runs.

We now systematically vary these hyperparameters to study the effect of agent capacity, training with Gumbel-Softmax with different temperatures. In particular, we study how agents' hidden sizes influence the complexity of NN systems. We note

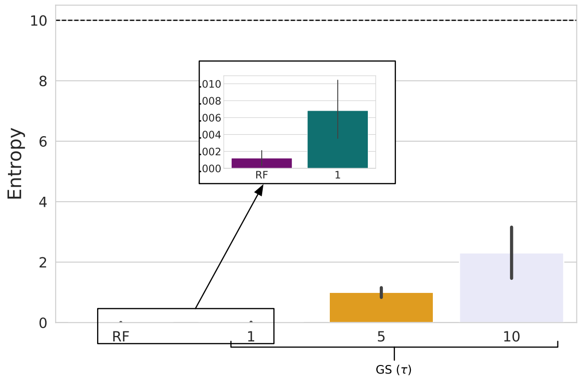

Fig. S8. Average entropy (in bits) of Speakers' outputs for different training regimes. The entropy is first averaged across the 330 outputs corresponding to all input color chips, then across Speakers in the considered training setting. Vertical bars represent standard deviation across Speakers. The horizontal line represents the entropy of a uniform distribution over 1024 categories (equal to $|V|$). The zoom box shows the low-entropy values (corresponding to (near-)discrete communication at training time).

h_a the hidden size of agent a, where $a = s$ if the agent plays the role of Speaker and $a = l$ if the agent is Listener. We vary h_s in $\{250, 500, 1000\}$ and h_l in $\{5, 10, 100, 500\}$. Across these experiments, we keep the same number of layers used in the main analysis. However, we observe the same pattern when varying the number of layers (not reported here). Finally, we only include successful runs in the analysis (discrimination accuracy strictly above 95%).

We start with the default Gumbel-Softmax temperature value $\tau = 1$, corresponding to an essentially discrete setup. Each experiment is repeated 20 times. Figure S9c shows that varying Listener's parameters does not impact the complexity of the emergent systems. On the other hand, in Figure S9a we observe that, with the same training regime, increasing h_s makes the systems significantly less complex ($p < 10^{-7}$, Kruskal-Wallis test). In short, when considering emergent system complexity, agent capacity only matters when it concerns the Speaker agent, that is, when the extra capacity occurs *before* the *discrete* communication channel bottleneck.

If h_s matters when communicating with a discrete channel, we look now at its effect when training with a smoother one, using $\tau = 10$. As noted in Supplementary 6, this large τ leads to fewer successful runs. We thus repeat this experiment 60 times. When varying Listener's capacity, agents were successful in the game only for $h_l \in \{5, 10\}$. For these successful settings, the average (standard deviation) complexities are 2.97 (0.31) and 2.87 (0.29) for h_l equal to 5 and 10 respectively, indicating no significant difference between naming-systems' complexities in this setting. The same observation holds when considering different Speaker's capacity with $h_s \in \{250, 500, 1000\}$. As shown in Figure S9b, in this more continuous setup there is no significant difference across Speakers with different capacities ($p = 0.07$, Kruskal-Wallis test).

A natural interpretation of these results is that, when the channel is virtually discrete (low τ), the complexity minimization pressure is so high that Speaker tries to compress the input as much as it can, passing only the information that is strictly necessary for communication success. The more capacity Speaker has, the better it succeeds at compactly encapsulating useful information about its inputs, resulting in *simpler* systems for *larger* Speakers. However, this effect disappears for the more continuous setup with high τ, as the minimization pressure due to discreteness is no longer at play.

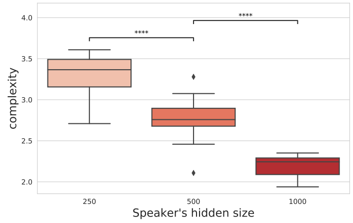

(a) Effect of Speaker's hidden size when τ=1: Systems' complexity with respect to Speaker hidden size. Results show a significant difference between the different hidden sizes with $p < 10^{-7}$ when using the Kruskal-Wallis test.

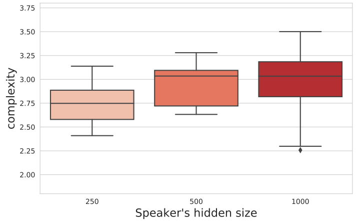

(b) Effect of Speaker's hidden size when τ=10: Systems' complexity with respect to Speaker hidden size. Results show *no* significant difference between the different hidden sizes with $p = 0.07$ when using the Kruskal-Wallis test.

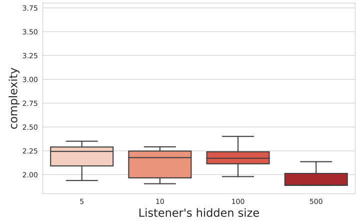

(c) Effect of Listener's hidden size when τ=1: Systems' complexity with respect to Listener hidden size. Results show *no* significant difference between the different hidden sizes with $p = 0.10$ when using the Kruskal-Wallis test.

Fig. S9. Systems' complexity for different agents' hidden sizes and different training regimes. Pairwise differences evaluated with Bonferroni-corrected Mann-Whitney-Wilcoxon (****p<0.0001); 'ns' differences are not labeled.

Kharitonov and colleagues (4) present further evidence of the interaction between discreteness and Speaker/Listener capacity in the context of a toy experiment. They show that, with a more continuous channel, both a larger Speaker and a larger

Listener can memorize a data-set with random input-label associations. With a discrete channel, however, an extra-capacity Speaker will memorize the labels and only transmit a compressed summary of the needed information. Increasing Listener's capacity, on the other hand, has no effect, as the agents are simply incapable to learn to transmit the high-complexity "raw data" for the Listener to process through the discrete channel.

9. How are color naming systems (in)efficient?

We saw in the main text that systems with high discriminative need tend to be more complex, but not inefficient, whereas, when we let the channel be more continuous, we see the emergence of systems that are more complex *and* inefficient. In this section, we explore one concrete way in which color-naming systems might be (in)efficient (without claiming that it is the only one). To this end, we introduce a measure that quantifies, for a word in a given system, the degree to which its denotation is also covered by another word, or how *separate* the meaning of a word is from that of the others. We refer to this measure as *sep* (for *separation*). Intuitively, in efficient naming systems all words should have very high *sep*. Lower-*sep* words are redundant leading to inefficient partition of the color space. Indeed, their presence increases the system's complexity ($I(M;W)$) with no notable increase in accuracy ($\propto KL[M||\hat{M}]$ with KL the Kullback–Leibler divergence).

A. Estimating sep. We aim to quantify how redundant/separate each word is. For example, in English, the word "scarlet" is in a sense redundant, as its meaning is included in that of "red". Using both words makes the color-naming system less efficient. Indeed, "red" is a fine word to refer to scarlet tonalities, and adding "scarlet" only slightly increases communication accuracy at the cost of an increase in complexity ("scarlet" might still be useful as a specialized word, of course). Formally, to measure if a word w is redundant, we need to find a w' that covers the same reference. To do so, we define $C_w = \{c, \text{ s.t. } c \text{ denoted by } w\}$ the set of colors/references denoted by the word w. Moreover, for two given words w and w', $p(C_w \not\subset C'_w)$, is the probability that the denotation of w is separate from that of w', such that:

$$\begin{aligned} p(C_w \not\subset C'_w) &= p(w) \times p(\overline{w'}|w) \\ &= p(w) \times (1 - p(w'|w)) \\ &= p(w) - \sum_c p(w, w'|c) \times p(c) \end{aligned} \quad [1]$$

Since, by construction, for any given color c, we sample words independently from Speaker, we have:

$$p(C_w \not\subset C'_w) = p(w) - \sum_c p(w|c) \times p(w'|c) \times p(c) \quad [2]$$

Finally, we search, among all possible words distinct from w, the word from which w is *least* likely to be separated (in terms of denotation). To this end, we define:

$$sep(w) = min_{w' \neq w} p(C_w \not\subset C'_w) \quad [3]$$

Low $sep(w)$ indicates that w is redundant, that is, it exists a w' that is likely to cover w's denotation. Figure S10 presents 3 scenarios with different *sep* values.[†]

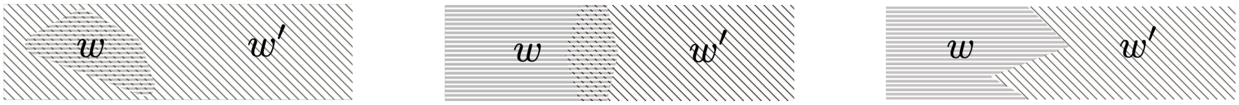

(a) No separation: $sep(w)$=0 **(b)** Partial separation: 0<$sep(w)$<1 **(c)** Perfect separation: $sep(w)$=1

Fig. S10. Three hypothetical two-word systems. The regions represent the extension of items (color chips) denoted by each word. Each system partitions the extension differently leading to different $sep(w)$ values.

B. Compared systems. We measure *sep* of the words in emergent NN systems when varying both discriminative need and channel smoothness. Based on the main paper results, we expect that, while high discriminative need complexifies the system, it should not impact the nature of its words, while a smooth channel might lead to the emergence of redundant words. In practice, we compare 3 different settings:

- `smooth`: composed of 5 different successful NN systems obtained with an intermediate discriminative need ($percentile$=50) and a largely smooth channel (τ=10).
- `need`: composed of 5 different successful NN systems arising when agents have an extreme discriminative need ($percentile$=20), and a discrete-like channel (τ=1).

[†] We verified that sep does not depend on the number of used words. In particular, FCM partitions till $K = 20$ comprise only separate words with $sep(w) \approx 1$, confirming the validity of our measure.

- `control`: a control setup containing 5 successful NN systems obtained with *percentile*=50 (similar to `smooth`) and τ=1 (similar to `need`).

Note that all studied systems allow successful communication (> 95% success rate in the discrimination game).

C. Results. Both the `need` and `control` settings avoid redundant words. In fact, Figures S11c and S11b show that, for any considered system, $sep(w) \approx 1$ for all w. On the other hand, `smooth` displays a different trend. We observe in Figure S11a that all systems in `smooth` (i.e., trained with high $\tau = 10$) include some words with low *sep*, confirming that a smooth channel leads to the emergence of redundant words, at the cost of efficiency.

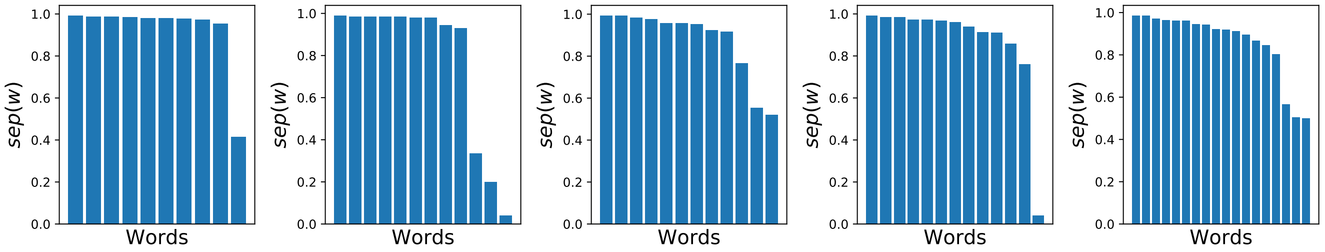

(a) The 5 systems of the *smooth* setting

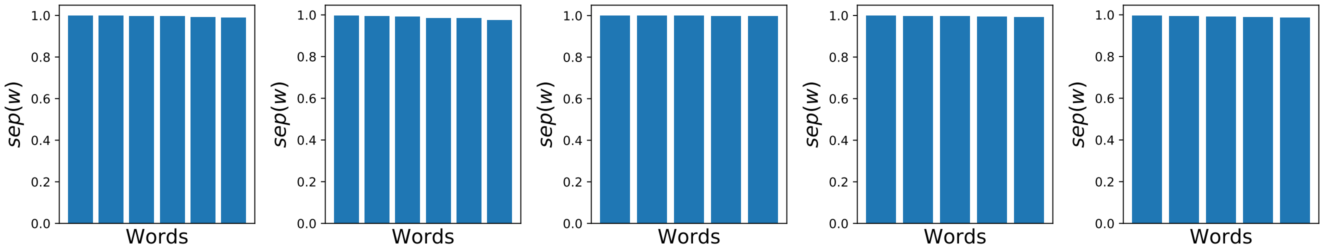

(b) The 5 systems of the *need* setting

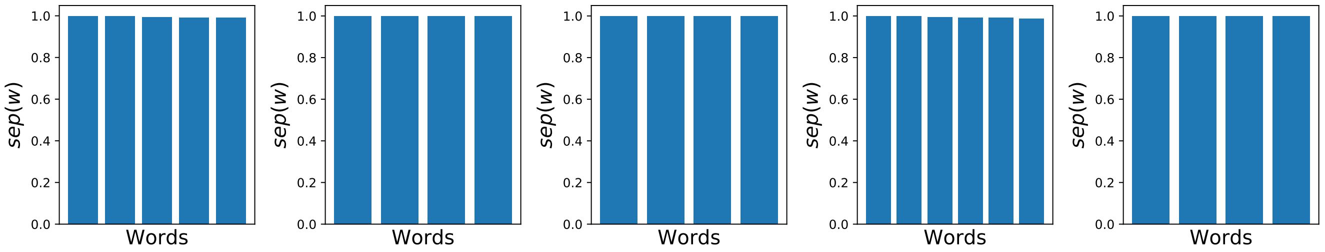

(c) The 5 systems of the *control* setting

Fig. S11. sep across different systems. The histograms denote *all* unique words of a system, sorted by decreasing sep. Each row represents one setting, and each sub-figure one system (out of 5) in the corresponding setting.

Our results emphasize the difference between the effect of complexification due to higher discriminative need vs. smoothness. A high discriminative need will complexify the naming system by introducing separate words. However, varying channel smoothness changes both system complexity and how redundant/separate its words are. That is, with a discrete channel (`need` and `control` settings), we observe the emergence of systems containing only separate words, whereas when agents communicate through a smooth channel (`smooth` setting), NN systems start to develop several redundant words.

Finally, *sep* can be related to the notion of *basic terms* introduced by Berlin and Kay (5). Berlin and Kay state that one condition for a term/word to be basic is to have *"its signification [...] not included in that of any other color term"*. Words meeting this condition will have high *sep*. Hence, we can relate the idea of a basic term to the notion of efficiency, in that systems where most words meet this condition will lead to an efficient color-naming system.

10. Direct comparison of color space partitions

The distribution of emergent NN naming systems along the accuracy and complexity axes is strikingly similar to that of the WCS natural languages. The relation between the specific way in which NN systems cluster the color space and the partitions created by human languages is more nuanced. NN systems share with human systems the fundamental property of partitioning

the color space into convex regions, but they do not rely on the dark/light dimension as the core axis along which to partition colors. Also, they appear to stay closer to a purely perception-based partition of color space than human languages do, which actually makes them *more* convex than human languages.

To quantify the similarity between NN and human color partitioning, we frame it as a clustering problem. The sets of colors denoted by the same name in a NN naming system are treated as clusters, and compared against ground-truth partitions provided by the WCS languages (we also discuss an experiment in which FCM clustering solutions are used as gold standards to compare both NN and natural languages against). We adopt the standard F_1 clustering quality evaluation measure (6, Ch. 16). F_1 takes its highest possible value of 1 when a clustering solution (determined, in our case, by how colors are grouped by name) is identical to the ground truth (in terms of how it partitions the color space). We discretize the NN naming distributions by labeling each color with the name maximizing $P(w|c)$. For human systems, we use majority names across subjects as color labels.

As there is a lot of variation in human language, no NN system could be similar to all WCS systems. Thus, for each NN system, we pick the largest F_1 score it attains when compared to all human systems of the same cardinality (that is, with the same number of color names after discretization). We refer to this score as *best F_1*. Best F_1 thus measures the degree to which a NN naming system is similar to at least one human system.

Comparison \ Cardinality	3	4	5	6	7
NN vs. WCS	0.559 (0.039)	0.472 (0.036)	0.521 (0.051)	0.547 (0.030)	0.531 (NA)
WCS vs. WCS	0.869 (0.031)	0.717 (0.036)	0.685 (0.060)	0.765 (0.081)	0.719 (0.090)
informed baseline	0.412 (0.023)	0.319 (0.044)	0.280 (0.034)	0.262 (0.053)	0.225 (0.044)
NN vs. FCM	0.675 (0.049)	0.610 (0.102)	0.604 (0.052)	0.611 (0.025)	0.602 (NA)
WCS vs. FCM	0.466 (0.022)	0.510 (0.056)	0.481 (0.043)	0.464 (0.063)	0.485 (0.064)

Table S2. Average best F_1 by cardinality (standard deviation in parenthesis; the latter is NA when there is only one tested naming system of the corresponding cardinality). *NN vs. WCS*: averages across NN naming systems compared to WCS languages as ground truth. *WCS vs. WCS*: averages across WCS languages, using nearest WCS language as ground truth. *Informed baseline*: for each WCS language, generate 100 pseudo-naming-systems by shuffling its names; pick best F_1 with original naming scheme across pseudo-naming systems; average best F_1s across languages with same cardinality. *NN vs. FCM*: averages across NN naming systems when using discretized FCM clustering solutions as ground truth. *WCS vs. FCM*: averages across natural language naming systems when using FCM solutions as ground truth. Since there is only one FCM solution per cardinality, in the last two comparisons average best F_1 equals average F_1.

The first row of Table S2 reports averaged best F_1 for the NN systems when using the WCS names as ground-truth labels.[‡] To make sense of these numbers, we compare them to an upper bound and a baseline in the next two rows. The upper bound is given by averaging the same score across WCS languages, when using the nearest language to each as ground truth. NN naming schemes are clearly farther away from those of the nearest natural languages than natural languages are from each other. The baseline is obtained by generating, for each WCS language, 100 pseudo-naming-systems with the same label frequencies. The F_1 score with respect to the reference WCS language is computed for each of the 100 pseudo-naming-systems, and the best one is retained for each WCS language. This is an *informed* baseline because it has access to the ground-truth label distribution. Across all cardinalities, NN systems are much closer to actual WCS languages than the informed baseline is.

Where does the difference between natural and NN naming schemes come from? A partial answer is provided by the next two rows of Table S2, where we evaluate to what degree natural and NN systems match the partitions obtained through fuzzy c-means (FCM) clustering (7) in CIELAB space. The latter should approximate color space partitions that are optimal on purely perceptual grounds (FCM returns partitions that minimize within-cluster distance in color space). We compare each NN/human naming system to the (discretized) FCM solution with K equal to the naming system cardinality.

The *NN vs. FCM* comparison reveals that NN systems are closer to the purely perception-based FCM partitioning than to natural languages (*NN vs. WCS* in the first row of the table). Natural languages in turn, as shown in the *WCS vs. FCM* row, are further away from FCM solutions than NN naming systems are. So, the difference between WCS and NN systems stems, at least in part, from the fact that human naming systems drifted further apart from purely perceptual pressures than NN systems did. This makes sense: human language evolution is subject to many external pressures (social, environmental, etc.) that are not part of our language emergence simulations.

Figure S12 provides a more qualitative insight into how NN and natural language color partitions differ, by visualizing emergent naming systems of cardinality 3 and 5 together with their nearest WCS languages. To avoid cherry-picking, we chose, for each cardinality, the naming systems with median best F_1. The results are generally representative, although for higher cardinalities a qualitative comparison becomes problematic due to considerable noise in the WCS data.

Wobé, the reference 3-color-term human system (top right of Figure S12), illustrates the near-universal 3-way split into "light", "dark" and red (5). The corresponding NN system (top left) does not encode the dark/light split. While unnatural in this respect, the partitioning is, like those found in human languages, clearly convex (8, 9). The NN system clusters correspond, moreover, to the other basic colors attested in low-complexity human languages, once we exclude the dark/light distinction: red, green and a yellow/brown patch.

[‡] We include all successful NN systems also used in the main experiments.

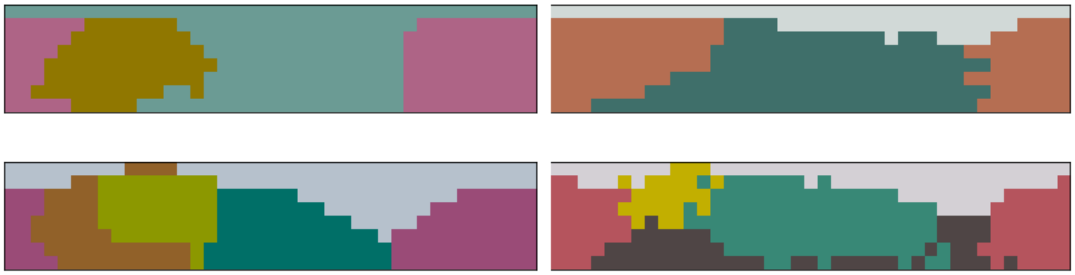

Fig. S12. Top: a 3-word NN naming system (left) compared to Wobé (Niger-Congo) (right), its closest WCS counterpart. Bottom: a 5-word NN system (left) compared to Bauzi (East Geelvink Bay) (right), its closest WCS counterpart. Same-color clusters represent colors denoted by the same word (after discretizing). Cluster colors are obtained by averaging the RGB values of all colors in the cluster. Refer to Figure 1 in the main article for a separate rendering of each chip, shown in the same arrangement.

Bauzi (bottom right of Figure S12) follows a typical 5-term naming scheme: white (light), black (dark), red, green and yellow. The corresponding NN system (bottom left) omits the black category, but does not radically depart otherwise from the human system, with the colors clustered into white, red, green and yellow areas. Again, the NN partition is clearly convex.

To quantitatively substantiate the qualitative claim about NN systems' convexity, we computed the *degree of convexity* of each NN system (and, for comparison, WCS and FCM systems) using the method recently proposed by Steinert-Threkeld and Szymanik (10, p. 5). For each (discretized) color name, we compute the ratio of number of points denoted by the name to the number of points in their convex hull, weighting by partition size and normalizing. Averaged results are given in Table S3.

Naming System \ *Cardinality*	3	4	5	6	7
NN	0.999 (0.003)	0.998 (0.004)	0.999 (0.002)	1.00 (0.000)	0.997 (NA)
WCS	0.964 (0.030)	0.936 (0.026)	0.935 (0.039)	0.949 (0.047)	0.926 (0.038)
FCM	0.991 (NA)	1.000 (NA)	1.000 (NA)	1.000 (NA)	1.000 (NA)

Table S3. Average degree of convexity by cardinality for different naming systems (standard deviation in parenthesis; the latter is NA when there is only one tested naming system of the corresponding cardinality).

The degree of convexity of NN systems is extremely high, and approaching that of FCM clustering (which naturally favors convexity because of its distance-minimizing objective). Remarkably, the degree of convexity of NN systems is *higher* than that of the natural languages in WCS. This might be due, again, to the fact that humans must optimize communicative constraints that are not entirely perception-driven, or, more simply, to the noise inherent in the WCS surveying methodology. We leave this intriguing question to further work.[§]

In sum, NN color-naming systems, like (and perhaps more than) human ones, show a clear tendency to partition the color space into convex regions. However, the latter regions depart to some extent from those typically defined by human color naming. NN systems might stay closer to a purely perceptual partitioning of the color space. Moreover, qualitatively, they do not seem to enforce the distinction between white (light) and black (dark), which is instead universally present in human languages. Note however that, as we report in Supplementary 5, NNs are in principle able to discover the dark/light distinction if we encourage it in the design of the game.

References

1. E Gibson, et al., Color naming across languages reflects color use. *Proc. Natl. Acad. Sci.* **114**, 10785–10790 (2017).
2. N Zaslavsky, C Kemp, T Regier, N Tishby, Efficient compression in color naming and its evolution. *Proc. Natl. Acad. Sci.* **115**, 7937–7942 (2018).
3. P Kay, CK McDaniel, The linguistic significance of the meanings of basic color terms. *Language*, 610–646 (1978).
4. E Kharitonov, R Chaabouni, D Bouchacourt, M Baroni, Entropy minimization in emergent languages in *Proceedings of ICML*. (virtual conference), pp. 2718–2728 (2020).
5. B Berlin, P Kay, *Basic color terms: Their universality and evolution.* (Univ of California Press), (1991).
6. C Manning, P Raghavan, H Schütze, *Introduction to Information Retrieval.* (Cambridge University Press, Cambridge, UK), (2008).
7. J Bezdek, *Pattern Recognition with Fuzzy Objective Function Algorithms.* (Kluwer, Boston, MA), (1981).
8. P Gärdenfors, *Conceptual Spaces.* (MIT Press, Cambridge, MA), (2000).
9. G Jäger, Natural color categories are convex sets in *Logic, Language and Meaning*, eds. M Aloni, H Bastiaanse, T de Jager, K Schulz. (Springer, Berlin, Germany), pp. 11–20 (2010).
10. S Steinert-Threlkeld, J Szymanik, Ease of learning explains semantic universals. *Cognition* **195**, 104076 (2020).

[§] Could the convexity of NN systems be an artefact of how we pick distractors based on a discrimination need threshold, thus indirectly favoring systems where nearer color chips get the same name (as they are likely to be below this threshold)? This is not the case, as shown by the fact that all 6 successful systems discussed in Supplementary 2, where there is *no* control on target-distractor distance, are also fully convex (degree of convexity uniformly at 100%).
